# Caveolin-1: An Oxidative Stress-Related Target for Cancer Prevention

**DOI:** 10.1155/2017/7454031

**Published:** 2017-05-04

**Authors:** Shengqi Wang, Neng Wang, Yifeng Zheng, Jin Zhang, Fengxue Zhang, Zhiyu Wang

**Affiliations:** ^1^Department of Mammary Disease, Discipline of Integrated Chinese and Western Medicine in Guangzhou University of Chinese Medicine, The Second Affiliated Hospital of Guangzhou University of Chinese Medicine, Guangzhou, Guangdong, China; ^2^The Research Center for Integrative Medicine, Guangzhou University of Chinese Medicine, Guangzhou, Guangdong, China; ^3^Post-Doctoral Research Center, Guangzhou University of Chinese Medicine, Guangzhou, Guangdong, China; ^4^Department of Breast Oncology, Sun Yat-Sen University Cancer Center, State Key Laboratory of Oncology in South China, Collaborative Innovation Center for Cancer Medicine, Guangzhou, Guangdong, China

## Abstract

Aberrant oxidative metabolism is one of the hallmarks of cancer. Reactive species overproduction could promote carcinogenesis via inducing genetic mutations and activating oncogenic pathways, and thus, antioxidant therapy was considered as an important strategy for cancer prevention and treatment. Caveolin-1 (Cav-1), a constituent protein of caveolae, has been shown to mediate tumorigenesis and progression through oxidative stress modulation recently. Reactive species could modulate the expression, degradation, posttranslational modifications, and membrane trafficking of Cav-1, while Cav-1-targeted treatments could scavenge the reactive species. More importantly, emerging evidences have indicated that multiple antioxidants could exert antitumor activities in cancer cells and protective activities in normal cells by modulating the Cav-1 pathway. Altogether, these findings indicate that Cav-1 may be a promising oxidative stress-related target for cancer antioxidant prevention. Elucidating the underlying interaction mechanisms between oxidative stress and Cav-1 is helpful for enhancing the preventive effects of antioxidants on cancer, for improving clinical outcomes of antioxidant-related therapeutics in cancer patients, and for developing Cav-1 targeted drugs. Herein, we summarize the available evidence of the roles of Cav-1 and oxidative stress in tumorigenesis and development and shed novel light on designing strategies for cancer prevention or treatment by utilizing the interaction mode between Cav-1 and oxidative stress.

## 1. Introduction

The cumulative production of reactive species, particularly reactive oxygen species (ROS) and reactive nitrogen species (RNS), through either endogenous or exogenous insults is termed oxidative stress [1]. Oxidative stress has been reported to be implicated in the etiology and progression of multiple human diseases, such as neurodegenerative disease, inflammatory disease, cardiovascular disease, allergies, immune system dysfunctions, diabetes, and aging as well as cancer [1]. Reactive species play an important role in cancer etiology and progression and are being progressively elucidated [2–4]. Physiological levels of reactive species are crucial for ensuring cell survival. However, overproduction of reactive species is detrimental to cells and could induce tumorigenesis by oxidizing specific intracellular moieties, resulting in genetic mutations and activations of ontogenetic pathways that stimulate proliferation and neoplastic transformation [2–5]. Cancer cells are usually submitted to higher levels of reactive species as a result of aberrations in oxidative metabolism (e.g., impaired mitochondrial oxidative phosphorylation [6] and increased aerobic glycolysis [7]) and signaling pathways (e.g., Ras [8] and AKT [9] activation), which further stimulate the malignant phenotypes of death evasion, angiogenesis, invasiveness, and metastasis [10]. Therefore, antioxidant therapies or dietary supplementations of antioxidants have gained considerable interests in cancer prevention [11–15]. In addition, cancer patients widely take antioxidant supplements in order to lower oxidative damages of chemotherapy and radiation therapies to normal tissues [16–19]. It is estimated that approximately 45–80% of breast cancer patients use antioxidant supplements after diagnosis or during breast cancer treatment [17]. Common antioxidants used by cancer patients include glutathione (GSH), *β*-carotene, vitamin A (retinoic acid), vitamin C (ascorbic acid), vitamin E, and selenium [11, 13, 14].

Caveolae are 50 to 100 nm flask-shaped invaginations of the plasma membrane which have important roles in membrane trafficking (endocytosis and transcytosis), maintaining membrane lipid composition and cell signal transduction [20]. Cav-1, the major structural protein of caveolae, functions as scaffolding protein [21] regulating multiple physiological processes including caveola biogenesis, vesicular transport, cholesterol homeostasis, and signal transduction [22]. Recently, Cav-1 has been demonstrated to be closely involved in tumorigenesis and development, affecting the proliferation [23, 24], survival [25, 26], apoptosis [27, 28], migration [29, 30], invasion [24, 31], metastasis [32, 33], autophagy [34, 35], transformation [36], anoikis [37, 38], and chemotherapy resistance [39, 40] processes of cancer cells. Cav-1 is generally regarded as a tumor suppressor, and studies have implicated loss of Cav-1 in the pathogenesis and progression of multiple human cancers [41–44]. For example, loss of Cav-1 in cancer-associated fibroblasts (CAFs) could result in an activated tumor microenvironment, thereby driving early tumor recurrence, metastasis, and poor clinical outcomes in breast cancer [45]. In contrast, overexpression of Cav-1 in CAFs predicted a good outcome [45]. On the other hand, Cav-1 is also regarded as an oncoprotein in some kinds of malignancies. It was reported that the expression of Cav-1 was related to poor prognosis in lung pleomorphic carcinoma [46] and prostate carcinoma [47, 48]. In addition, Liu's group demonstrated that Cav-1 exhibited a stage-dependent, functional fluctuation during the progression of pancreatic cancer [49].

Recently, increasing evidences have showed that the oxidative stress processes in cancer cells were closely associated with Cav-1 [50–52]. Cav-1 is suggested to be a potential oxidative stress-related target during oxidative stress-induced cancer initiation and development. Reactive species overproduced in oxidative stress processes could modulate the expression [53], degradation [54], posttranslational modifications [55–57], and membrane trafficking [58] of Cav-1. Meanwhile, Cav-1 also has a feedback regulation effect on oxidative stress status in tumor microenvironment [50, 52]. For example, Cav-1 could attenuate hydrogen peroxide-induced oxidative damages to lung carcinoma cells [52]. More importantly, emerging evidences have indicated that multiple antioxidants could exert antitumor activities in cancer cells and protective activities in normal cells by modulating the Cav-1 pathway [59–61]. For example, treatment with antioxidants (such as quercetin, N-acetyl-cysteine, and metformin) was reported to reverse Cav-1 loss-induced phenotypes, such as mitochondrial dysfunction, oxidative stress, and aerobic glycolysis in CAFs [60]. Altogether, these findings indicate that Cav-1 may be a promising oxidative stress-related target for cancer antioxidant prevention. Considering the critical role of oxidative stress in mediating the therapeutic efficacies of radiotherapy and chemotherapy, understanding the action mechanisms of Cav-1 during oxidative stress processes is important for improving the clinical outcomes and for developing novel diagnostic tools and drugs for cancer antioxidant prevention. Herein, we summarize the current evidence of the roles of Cav-1 and oxidative stress, as well as their interaction in tumorigenesis and progression.

## 2. Structure, Localization, and Biofunctions of Cav-1

**Figure 1:**
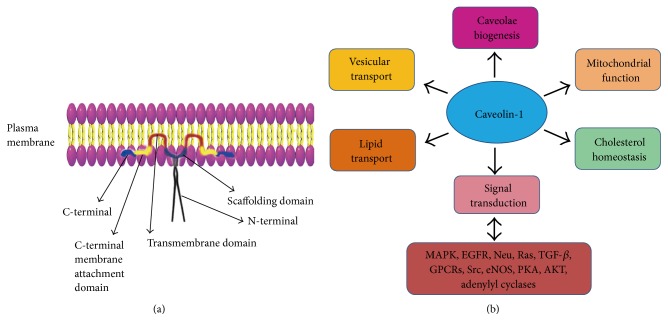
Structure and biofunctions of Cav-1. (a) The structure of Cav-1. The C-terminal, the C-terminal membrane attachment domain, and the transmembrane domain of Cav-1 are highlighted in blue, yellow, and red, respectively. The Cav-1-scaffolding domain (CSD) is responsible for Cav-1 interaction with other molecules. (b) Cav-1 biofunctions. Cav-1 is involved in the regulation of multiple physiological processes including caveola biogenesis, vesicular transport, cholesterol homeostasis, lipid transport, mitochondrial function, and signal transduction.

Originally named to describe the cave-like invaginations on the cell surface, caveolae are 50–100 nm membrane microdomains which represent a subcompartment of the plasma membrane and are enriched in glycosphingolipids, cholesterol, and lipid-anchored membrane proteins [22, 62]. Caveolins are caveolae-resident structural and scaffolding proteins, which are critical for the formation of caveolae and their interaction with signaling entities [22]. Each caveola contains approximately 100 to 200 caveolin molecules. There are three isoforms of caveolins (Cav-1, Cav-2, and Cav-3), while Cav-1 is the principal component of caveolae. Cav-1 (21–24 KDa) is an integral membrane protein with an unusual hairpin-like conformation (Figure 1(a)). The N- and C-terminal cytoplasmic tails of Cav-1 are separated by a hydrophobic segment that does not cross the membrane [63–65]. Both the C- and N-termini face the cytoplasm [66]. Cav-1 oligomerizes through amino acids 1–101. This oligomerization region contains the scaffolding domain (juxtamembrane 82–101 amino acids). Cav-1 has been identified primarily as two isoforms, Cav-1*α* and Cav-1*β*, which differs in their N-termini and derives from alternative translation initiation sites [67]. The presence of Cav-1 has been verified in most cell types by biochemical and morphological experiments, including epithelial cells, endothelial cells, fibroblasts, smooth muscle cells, and adipocytes [22, 68, 69]. Although Cav-1 is expressed ubiquitously, the levels of its expression vary among different tissues [22]. Cellular organelles where Cav-1 is enriched include the mitochondrion, the nucleus, the Golgi complex, and the endoplasmic reticulum [21, 22].

As shown in Figure 1(b), Cav-1 is involved in the regulation of multiple physiological processes, including caveola biogenesis, vesicular transport, cholesterol homeostasis, lipid transport, mitochondrial function, and signal transduction [22]. Cav-1 is a molecular hub. It could integrate transduction and negatively regulate a variety of signaling molecules, which include mitogen-activated protein kinases (MAPKs), epidermal growth factor receptor (EGFR), Neu, Ras family GTPases, transforming growth factor-*β* (TGF-*β*), G-protein-coupled receptors (GPCRs), Src family kinases, endothelial nitric oxide synthase (eNOS), adenylyl cyclases, protein kinase A (PKA), and AKT [22, 70–74]. The caveolin-signaling hypothesis proposes that the interactions between Cav-1 and these molecules are through the Cav-1-scaffolding domain (CSD) in Cav-1 molecule [72]. The Cav-1-scaffolding domain is also necessary for direct binding to cholesterol which participates in cholesterol transport and raft organization [75–77].

## 3. Emerging Role of Cav-1 in Cancer Prevention

As stated above, Cav-1 is a molecular hub-integrating transduction of multiple cellular molecules which are closely connected with the biological behaviors of cancer cells [78]. In addition, Cav-1 participates in multiple protein/protein and protein/lipid interactions which are critical for cell survival [22]. Undoubtedly, dysfunctional Cav-1 plays an important role in tumorigenesis and progression [79]. Human *CAV1* gene is localized to a suspected tumor suppressor locus (7q31.1) [53], which is a fragile genomic region and often deleted in cancers, suggesting that Cav-1 is possibly a tumor suppressor [80, 81]. The caveolin-signaling hypothesis proposes that Cav-1 could directly interact with multiple cancer-related signaling molecules including EGFR, Neu, TGF-*β*, Src, and AKT via the scaffolding domain and negatively modulate their aberrant activations. This hypothesis may further explain the role of Cav-1 as a tumor suppressor [22, 70]. Recent studies have implicated Cav-1 loss in the pathogenesis of various types of human malignancies [82–94] (Table 1). In breast cancer, loss of stromal Cav-1 was identified as a predictive biomarker of early tumor recurrence, lymph node metastasis, and tamoxifen-resistance as well as decreased survival in human breast cancer patients [42, 43, 95, 96], suggesting that Cav-1 functions as a tumor suppressor in breast cancer. Similar work confirmed that stromal Cav-1 loss was specifically associated with early ductal carcinoma in situ (DCIS) progression to invasive breast cancer, with shorter time to recurrence and higher recurrence rate [97]. In addition, Cav-1 is also negatively associated with breast cancer cells' transformation. Xie's group reported that Cav-1 expression was significantly attenuated in 3-phosphoinositide-dependent protein kinase-1- (PDK1-) mediated transformation of mammary epithelial cells [36]. Importantly, the prognostic value of stromal Cav-1 loss in breast cancers has now been independently validated [98–101] and has been extended to multiple types of human cancers, such as colorectal cancer [44], advanced prostate cancer [102], metastatic melanoma [41], gastric cancer [103], and osteosarcoma [104]. In colorectal cancer, it has been reported that stromal Cav-1 loss predicted poor survival [44]. In advanced prostate cancer, an absence of stromal Cav-1 was associated with metastatic disease and epithelial AKT activation [102]. In metastatic melanoma, stromal Cav-1 loss predicted poor survival of malignant melanoma patients [41]. In gastric cancer, epithelial Cav-1 loss could promote malignant progression of gastric cancer, and Cav-1 loss in CAFs heralded worse outcome of gastric cancer patient, suggesting Cav-1 level in CAFs may be a candidate therapeutic target and a useful prognostic marker of gastric cancer [103]. In osteosarcoma, Cav-1 downregulation could unleash c-Src and Met signaling, enabling osteosarcoma cells to invade neighboring tissues [104]. Conversely, overexpression of Cav-1 in osteosarcoma cell lines brought reduced malignancy with inhibited anchorage-independent growth, migration, and invasion. In addition, Cav-1 has also been reported to be closely connected with the multidrug resistance (MDR) of cancer cells [105–108]. For example, it was reported that Cav-1 could sensitize cancer cells to apoptosis in response to death stimuli, and a decrease of Cav-1 expression level was proved to contribute to chemotherapeutic cisplatin and carboplatin resistance [109].

**Figure 2:**
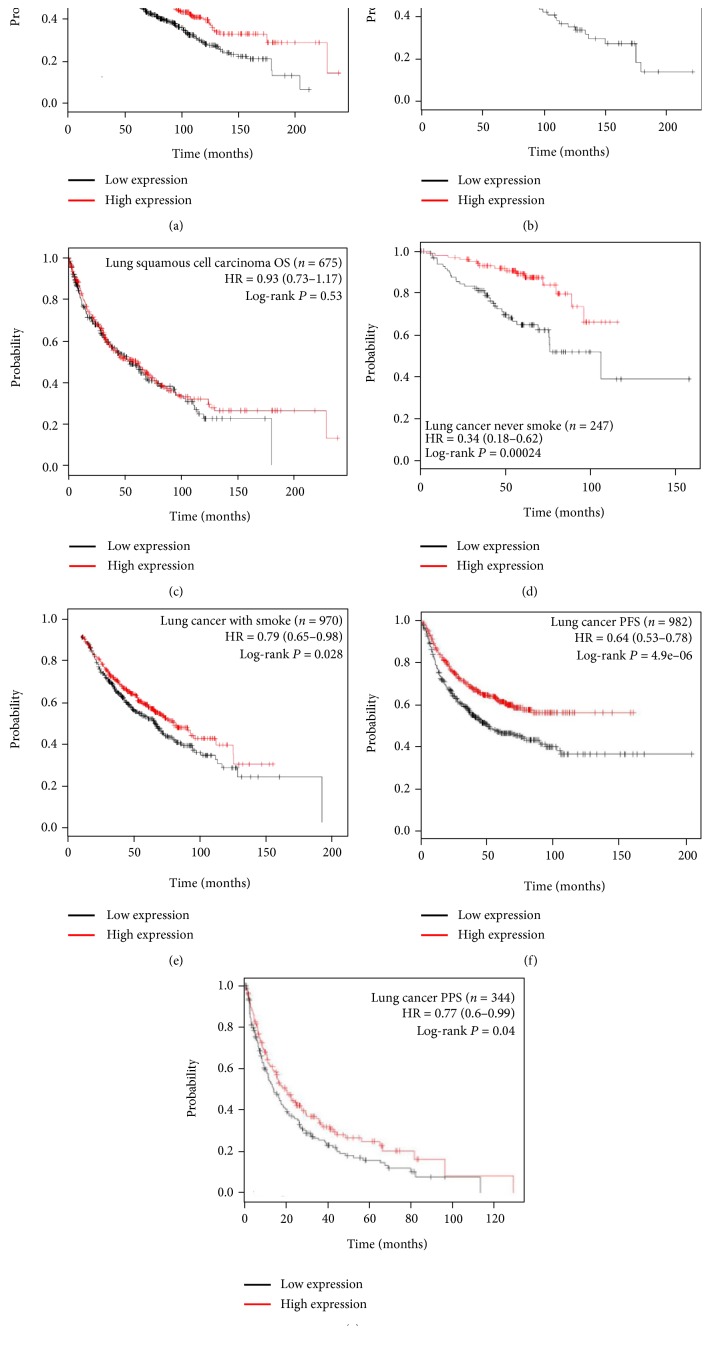
Tumor suppressive role of Cav-1 in lung cancer by Km-Plot analysis. (a) Cav-1 predicts an improved OS among lung cancer patients; (b-c) Cav-1^high^ expression is closely correlated with improved OS in lung adenocarcinoma but not in squamous carcinoma; (d-e) the clinical significance of Cav-1 in predicting OS is independent of smoke history; (f-g) besides OS, Cav-1^high^ expression is also significantly correlated with improved PFS and PPS.

Multiple clinical studies also verified the critical role of Cav-1 for cancer prevention. Bertino et al. observed that higher Cav-1 levels in tumor-associated stroma were significantly correlated with improved partial response rates (*P* = 0.036) and overall survival (OS) (*P* = 0.008) in advanced NSCLC patients, who treated with nanoparticle albumin-bound (nab) paclitaxel plus with carboplatin [110]. Furthermore, in a large cohort of 178 patients with colorectal cancer, Zhao et al. observed that the loss of stromal Cav-1 expression was associated with shorter disease-free survival (*P* = 0.000) and shorter OS (*P* = 0.000). This study further showed that the expression level of stromal Cav-1 was closely associated with histological type (*P* = 0.022), pathologic tumor-node-metastasis stage (*P* = 0.047), pathologic N stage (*P* = 0.035), and recurrence (*P* = 0.000) of colorectal cancer [44]. In a larger cohort of 724 patients with prostate cancer, Ayala and colleagues observed significantly decreased levels of stromal Cav-1 in concordance with increased Gleason score (*P* = 0.012) and reduced relapse-free survival (*P* = 0.009) [111]. Moreover, Jia et al. analyzed a total of 110 patients with esophageal squamous cell carcinoma and found that downregulation of stromal Cav-1 expression was associated with shorter disease-free survival (*P* < 0.001) and OS (*P* < 0.001), accompanied with more lymph node metastases (*P* = 0.020) and more local regional recurrences (*P* = 0.002) [112]. In addition, Yang et al. reported that increased Cav-1 expression was associated with prolonged overall survival rate in hepatocellular carcinoma (*P* = 0.021) [113]. Similar findings were also confirmed on breast cancer [99] and gastric cancer patients [114]. In particular, our analysis also revealed a close link between Cav-1 and lung cancer. Km-Plot analysis shows that lung cancer patients with Cav-1^high^ expression exhibit a better (OS) compared to those patients with Cav-1^low^ expression (Figure 2(a)). Meanwhile, high Cav-1 expression also indicates a better OS for lung adenocarcinoma but not for squamous cell lung carcinoma (Figures 2(b) and 2(c)). What is more, in both lung cancer patients with smoke history or no, Cav-1 exhibits a positive correlation with better OS, indicating that Cav-1-targeting strategy might be effective for lung cancer patients independent of the factor of smoke (Figures 2(d) and 2(e)). Interestingly, Cav-1^high^ expression also indicates an improved progression-free survival (PFS) and postprogression survival (PPS), further indicating the tumor suppressive role of Cav-1 in lung cancer (Figures 2(f) and 2(g)). In sum, accumulating evidence has indicated that Cav-1 might act as a tumor suppressor in multiple malignant tumors.

Interestingly, Cav-1 also acts as an oncoprotein depending on the tumor type and/or tumor stage. Particularly, Cav-1 seems to act as a tumor suppressor at early stages of cancer progression but as an oncoprotein in advanced-stage cancer [115]. For example, Cav-1 was found upregulated in multidrug-resistant colon cancer cells, adriamycin-resistant breast cancer cells, and taxol- and gemcitabine-resistant lung cancer cells [108, 116, 117]. Additionally, it was reported that *CAV1* silencing could sensitize breast cancer stem cells (CSCs) by limiting their self-renewal ability but promoting the differentiation process [118]. The conflict roles of Cav-1 in tumor progression may be partly explained by the observation that Cav-1 has several peptide domains with opposing functions [83]. Tyrosine phosphorylation, serine phosphorylation, and dominant-negative point mutations in these domains serve to functionally inactivate the tumor suppressor function of Cav-1 [83]. Meanwhile, we previously proposed a Cav-1 fluctuation model during cancer development [119]. It is suggested that Cav-1 might act as a kind of stress signal, which protects cells from hazardous damage, but its loss may make cells more sensitive to oncogenic events. However, when cancer progresses into the advanced stage or are treated with cytotoxic agents, the expression of Cav-1 would be upregulated to protect cancer cells escaping from death by speeding aerobic glycolysis, increasing stem cell population or overexpressing ABC transporters. Therefore, it is easy to understand current confusing evidence of Cav-1 in cancer development, and Cav-1 resurrection strategy is promising for preventing normal cells from malignant transformation.

## 4. Critical Role of Oxidative Stress Signaling in Mediating Cav-1 Anticarcinogenic Activities

Although physiological levels of reactive species function as important signaling of certain subcellular events such as enzymatic activity [5], gene expression [120], and protein synthesis [121], elevated levels of reactive species could initiate multiple toxic oxidative reactions including initiation of lipid peroxidation, direct inhibition of membrane sodium/potassium ATPase activity and mitochondrial respiratory chain enzymes, inactivation of glyceraldehyde-3-phosphate dehydrogenase, and membrane sodium channels [2–4]. All these toxicities are reported to play a role in carcinogenesis. In addition, elevated levels of reactive species could alter and damage many intracellular molecules, including nucleic acids, proteins, lipids, and polysaccharides [122], and therefore initiating a series of pathological processes and diseases. For example, reactive species could cause nicks in DNA, as well as malfunctions in the DNA repair mechanism. DNA oxidation induced by these reactive species generates 8-hydroxy-2′-deoxyguanosine, a product that is able to generate mutations in DNA and enhances carcinogenesis [123]. Furthermore, cancer cells usually exhibit increased levels of reactive species [124], which were found to facilitate cancer growth through sustained proliferation, apoptosis resistance, death evasion, angiogenesis, invasiveness, metastasis, and hypoxia-inducible factor 1 (HIF-1) activation [125–129]. Therefore, eliminating elevated oxidative stress is considered as an important strategy for cancer prevention.

**Figure 3:**
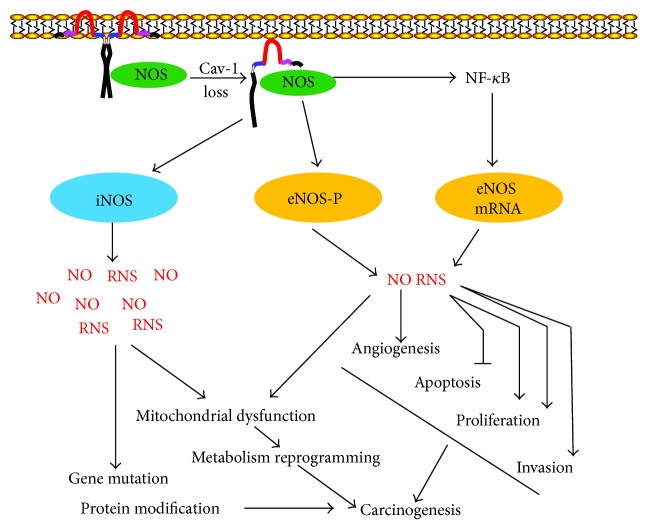
Cav-1 loss facilitates carcinogenesis through activating NOS activity. Cav-1 could directly interact with NOS enzymes through the scaffolding region. Following Cav-1 loss, eNOS will be released from the complex and activated through phosphorylation and mRNA overexpression, leading to the overproduction of NO and RNS. The high levels of NO and/or RNS would facilitate cell proliferation, apoptosis evasion, angiogenesis, and EMT process and finally induce carcinogenic transformation. Besides eNOS, iNOS will also be activated following Cav-1 loss. Since iNOS is capable of generating the micromolar level of NO, its activation will bring RNS burst and results in DNA damage and mitochondrial dysfunction, which finally promotes carcinogenesis.

Recent studies also revealed the critical role of oxidative stress in Cav-1 loss-mediated carcinogenic process. *CAV1* silencing in fibroblasts promoted ROS production, despaired mitochondrial activity, and DNA damage [50]. Similarly, treatment of fibroblasts with pro-oxidant buthionine sulfoxide caused the dose-dependent downregulation of Cav-1 [130]. In a coculture system, *CAV1^−/−^* fibroblasts were validated to promote breast cancer growth via driving aerobic glycolysis. Remarkably, the tumor-promoting effects of *CAV1*^−/−^ fibroblasts were reverted by recombinant overexpression of superoxide dismutase 2 (SOD2), thus implying that oxidative stress is critical for facilitating Cav-1 loss-induced carcinogenesis [131]. How does Cav-1 loss promote oxidative stress and mitochondrial dysfunction? Previous studies have found that Cav-1 loss has a close link with the increased NO synthase (NOS) activity and NO production (Figure 3). Although physiological NO plays significant role in maintaining cellular normal functions and modulating inflammatory response, excessive NO production is deleterious, as it inhibits mitochondrial OXPHOS chain and induces the overproduction of superoxide anion, hydrogen peroxide, and peroxynitrite, which exert potent cytotoxic and carcinogenic effects. For example, peroxynitrite could lead to mutation in the *p53* tumor suppressor gene and initiate oncogenic events [132]. NO is synthesized by at least four isoforms of NOS enzyme: endothelial NOS (eNOS), inducible NOS (iNOS), neuronal NOS (nNOS), and, more recently, mitochondrial NOS. Mounting evidence has validated the increased NOS expression and/or activities, especially for the eNOS subtype, in human malignancies such as breast, central nervous system, and colon tumors [133]. eNOS was demonstrated to produce nanomolar level of NO, and its activation was validated to promote cancer angiogenesis cascade, apoptosis evasion, and epithelial-mensenchymal transition (EMT) process [134]. Interestingly, eNOS was demonstrated to directly interact with Cav-1 through its scaffolding domain [135]. Meanwhile, Cav-1 internalization has been shown to further regulate eNOS activation [136]. *CAV1*-deficient mice also displayed elevated eNOS expression and phosphorylation, and eNOS inhibitor could block Cav-1 loss-induced cancer growth, angiogenesis, and metastasis [137]. Since only iNOS produces micromolar NO concentrations, iNOS activation would bring much more influences on DNA damage and mitochondrial activity. Therefore, targeting iNOS for cancer prevention and treatment has also been extensively studied. iNOS expression was also revealed upregulated in multiple malignancies including colon, breast, prostate, bladder, and skin tumors [138]. Meanwhile, administration of iNOS selective inhibitor was also demonstrated to interrupt the development of AOM-induced aberrant crypt foci, accompanied by reduced expression of COX-2 and oxidative stress [139]. All these findings highlighted the significant role of NOS/NO signaling in mediating Cav-1 anticarcinogenic activities.

Besides NOS/NO signaling, genetic profiling assay also showed that silencing *CAV1* in stromal cells, 48 known ROS-related genes were upregulated, which are mainly associated with mitochondrial oxidative phosphorylation and peroxisome biogenesis. Furthermore, 45 HIF-target genes, 21 glycolytic enzymes, and 86 NF-*κ*B-related genes were transcriptionally upregulated, implying that cellular metabolism was switched to glycolytic phenotype, which favors cancer initiation and growth [50]. ROS overproduction is closely associated with DNA damage response such as PARP. It was found that 5 PARP genes and 2 DNA-damage-induced transcripts Ddit3/Ddit4l were upregulated following CAV-1 knockdown [50]. Another genomic analysis also suggested that *CAV1*^−/−^ stromal cells showed the upregulation of 55 genes associated with oxidative stress, accompanied with overexpression of 129 genes correlated with DNA damage/repair response [50]. All these results indicated that elevated ROS production following *CAV1* silencing might facilitate cancer formation through activating DNA damage response. It is interesting to elucidate the precise interaction mode between Cav-1 and oxidative stress.

## 5. Cav-1 and Oxidative Stress Regulation

**Figure 4:**
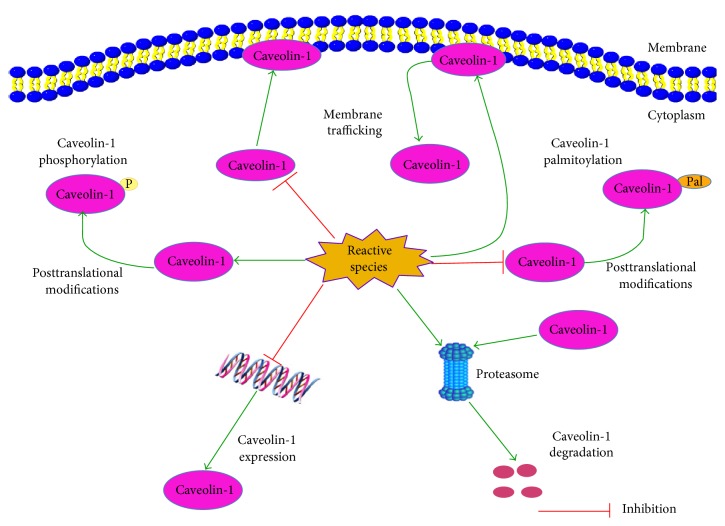
Cav-1 is an oxidative stress-related protein. On one hand, reactive species could decrease Cav-1 expression and induce its degradation through the proteasome pathway. On the other hand, reactive species could induce the phosphorylation but inhibit the palmitoylation of Cav-1. In addition, reactive species could induce Cav-1 internalization and inhibit the trafficking of newly synthesized Cav-1 to membrane raft domains.

Increasing studies have proved that Cav-1 is an oxidative stress-related protein, as reactive species could affect the expression, degradation, posttranslational modifications, and membrane trafficking of Cav-1 (Figure 4). Meanwhile, modulating the Cav-1 pathway could significantly affect the oxidative stress status in normal and cancer cells. Therefore, Cav-1 may be a target of antioxidants in oxidative stress modulation and cancer antioxidant prevention. Herein, we summarize the available evidences about the implications of Cav-1 in the oxidative stress modulation effect of antioxidants and shed novel insights for Cav-1-targeted antioxidant therapy in cancer.

### 5.1. Cav-1 Is an Oxidative Stress-Related Protein

Intracellular reactive species are generated to serve as the second messengers, and some are linked to caveolae-signaling systems. Numerous cell surface receptors, which initiate a signal transduction cascade involving reactive species when activated by ligand binding, are recruited in caveolae [69]. These membrane microdomains, which play a pivotal role in signal transduction [140], therefore have been proposed to be a preferred site of reactive species generation [141].

#### 5.1.1. Oxidative Stress Modulates the Expression and Degradation of Cav-1

Cav-1 reduction could significantly perturb the function of caveolae. In spite of Cav-1 reduction, caveolae are still able to be assembled at the plasma membrane, but their functions will be significantly impaired [142]. Oxidative stress could lead to the reduction of Cav-1 by modulating its expression and degradation (Figure 4). For example, Cai et al. reported that inhibitors of ROS could increase the expression of Cav-1 in human brain tumor microvascular endothelial cells [143]. Zhang et al. reported that high concentrations of glucose could decrease Cav-1 expression in lens epithelial cells [144]. Furthermore, Mougeolle and colleagues reported that hydrogen peroxide at nontoxic concentrations could increase the concentrations of reactive species in myoblasts and decrease the expression level of Cav-1. However, this phenomenon was not observed in the presence of a proteasome inhibitor, suggesting that Cav-1 was rapidly degraded by the proteasome [142]. Moreover, Luanpitpong and colleagues have found that superoxide anion and hydrogen peroxide could attenuate the expression of Cav-1 in lung carcinoma H460 cells [145]. Further mechanism studies indicated that the downregulation effect of superoxide anion and hydrogen peroxide on Cav-1 is modulated through a protein degradation mechanism via the ubiquitin-proteasome pathway [145].

#### 5.1.2. Oxidative Stress and Posttranslational Modifications of Cav-1

Cav-1 is mainly subject to two kinds of posttranslational modifications that regulate its activity, including phosphorylation and palmitoylation. Phosphorylation of Cav-1 is closely connected with cell apoptosis and cell attachment during oxidative stress. Increased expression of p-Cav-1 is antiapoptotic and may promote cell survival after oxidative stress [146, 147]. Cav-1 was first identified as a phosphoprotein in Rous sarcoma virus-transformed chicken embryo fibroblasts, which led to the hypothesis that Cav-1 may be a critical target during cellular transformation [148]. Since then, Cav-1 has been commonly identified as a phosphoprotein [56, 149–151]. Normally, Cav-1 is phosphorylated at a low [149, 150] or undetectable level [56, 151] in unstimulated cells. Reactive species generation could promote Cav-1 phosphorylation (Figure 4) [152]. For example, Volonte et al. suggested that multiple cellular stresses including high osmolarity, hydrogen peroxide, and UV light could induce the tyrosine 14-phosphorylation of Cav-1 [56]. Sun's group reported that ROS overproduction induced by high glucose-containing medium could time dependently increase Cav-1 phosphorylation in podocytes [153]. In addition, it was reported that an increased tyrosine-14 phosphorylation of Cav-1 was detected in human umbilical vein endothelial cells (HUVECs) after treatment with 100 *μ*M hydrogen peroxide for 30 minutes [154]. This increased tyrosine phosphorylation could be inhibited by tyrosine kinase inhibitors (herbimycin or genistein), which is consistent with the finding of Volonte [56]. What is more, Cav-1 could also be phosphorylated on serine-80 [155], and recent evidences suggested that serine phosphorylation of Cav-1 might be inversely correlated to tyrosine 14-phosphorylation [156].

Palmitoylation is the regulated, posttranslational modification of cysteine, serine, or threonine residues by the saturated fatty acid palmitate (e.g., palmitic acid), through a covalent thioester linkage [157]. Palmitoylation enhances the hydrophobicity of proteins and contributes to their membrane association. Palmitoylation typically occurs in membrane proteins and plays an important role in subcellular trafficking of proteins between membrane compartments, as well as in modulating protein-protein interactions [158]. Like many caveolae-targeted proteins, Cav-1 could also be acylated. Three cysteine residues near the C-terminus of Cav-1 (cysteines 133, 143, and 156) are susceptible to palmitoylation [159]. Cav-1 palmitoylation is required for cholesterol binding and transport to caveolae and for interaction with c-Src [160, 161]. Mutation of the cysteine residues could impair Cav-1 interaction with other acylated proteins [161] and its binding and transport of cholesterol. It has been reported that reactive species, such as hydrogen peroxide, could markedly inhibit the palmitoylation of Cav-1 (Figure 4) [150]. Moreover, the effect of hydrogen peroxide on Cav-1 palmitoylation usually occurs at much lower concentrations than those required to affect Cav-1 phosphorylation, indicating that the effect on palmitoylation is not a direct consequence of the increased phosphorylation.

#### 5.1.3. Oxidative Stress and the Membrane Trafficking of Cav-1

The function and activity of Cav-1 are tightly regulated by posttranscriptional modification as well as its subcellular localization. An early recognition that Cav-1 may be a sensitive target of oxidative stress modulation originated from the observation that oxidation of caveolar cholesterol by cholesterol oxidase could cause a reversible translocation of Cav-1 from the caveolae to the Golgi apparatus [162]. By applying immunofluorescence assay, Kang et al. have demonstrated that an exposure to high doses (1 mM) of hydrogen peroxide could induce Cav-1 internalization in NIH3T3 fibroblasts [58]. Similarly, by carrying out biochemical experiments, Blair's group also found that treatment of endothelial cells with oxidized LDL, but not native LDL, could lead to the translocation of Cav-1 from plasma membrane caveolae to an intracellular membrane fraction [163]. In addition, metabolic labeling experiments also showed that hydrogen peroxide could inhibit the trafficking of newly synthesized Cav-1 to membrane raft domains in endothelial cells [150], and impairment of Cav-1 synthesis by hydrogen peroxide was not responsible for the diminished trafficking. Altogether, these results indicate that oxidative stress could affect the membrane trafficking of Cav-1 (Figure 4).

### 5.2. The Regulation Effects of Cav-1 on Oxidative Stress

**Figure 5:**
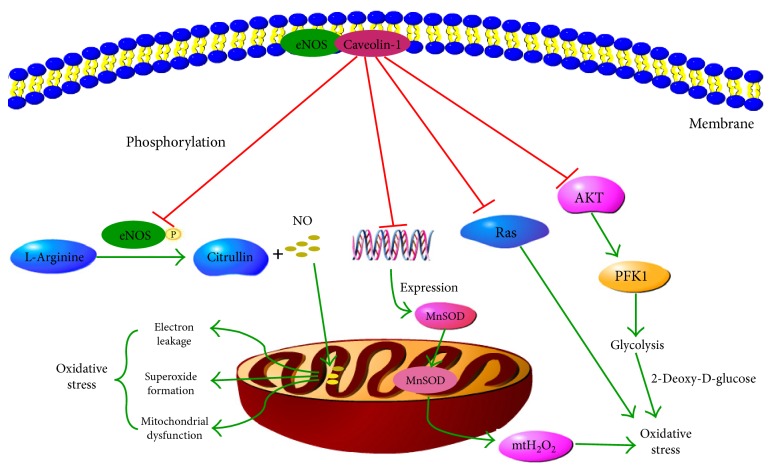
Regulation mechanisms of Cav-1 on oxidative stress. eNOS is maintained in an inactive state in caveolae by its interaction with Cav-1-scaffolding domain. Upon stimulation, eNOS is released and phosphorylated and could catalyze the NO synthesis reaction. NO overproduction could result in the inhibition of mitochondrion by attenuating the terminal phase of the electron transport chain complex, leading to electron leakage, superoxide formation, and mitochondrial dysfunction. Furthermore, Cav-1 could inhibit the transcription of MnSOD and thus reduce the release of excess mitochondria-derived H_2_O_2_ (mtH_2_O_2_). Moreover, glycolysis process could lead to the overproduction of reactive species, while 2-deoxy-D-glucose, a glycolysis inhibitor, could prevent reactive species burst. Phosphofructokinase 1 (PFK1) is the rate-limiting enzyme of the glycolysis process. Cav-1 could inactivate the PI3K/AKT/PFK1 pathway and therefore block the glycolysis process. Finally, Cav-1 could prevent the overproduction of reactive species by inactivating the Ras pathway.

Cav-1 also has a feedback regulation effect on oxidative stress status in cells (Figure 5). Shiroto and colleagues found that *CAV1* silencing in endothelial cells could increase the production of ROS in mitochondria and induce oxidative stress [164]. 2-deoxy-D-glucose, a glycolytic inhibitor, attenuated this increase, suggesting Cav-1 is in control of oxidative stress through glycolytic modulations [164]. Pavlides and colleagues have demonstrated that Cav-1 loss could induce oxidative stress, mimic hypoxia, and drive inflammation in the tumor microenvironment [50]. Martinez-Outschoorn et al. found that Cav-1 loss could lead to mitochondrial dysfunction, oxidative stress, and aerobic glycolysis in cancer-associated fibroblasts [60]. Wang et al. reported that Cav-1 knockdown resulted in eNOS redistribution to the perinuclear region and nearly doubled insulin-induced NO production in vascular endothelial cells [165]. Since NO is a competitive inhibitor of oxygen in the cytochrome oxidase present in mitochondrial complex IV, increased NO production could result in the inhibition of mitochondrion by attenuating the terminal phase of the mitochondrial electron transport chain complex, leading to electron leakage, superoxide formation, and mitochondrial dysfunction (Figure 5).

Cav-1 is highly expressed in endothelium. Therefore, the enriched Cav-1 in tumor vessels provides an interesting opportunity for Cav-1-targeted therapies. Gratton et al. demonstrated that a cell-permeable peptide derived from the CSD of Cav-1 could regulate microvascular permeability via inhibiting eNOS, and consequently markedly reduced tumor progression in mice [166]. Similarly, Suchaoin et al. reported that lung cancer H460 cells stably transfected with *CAV1*-overexpressing plasmids (H460/Cav-1) exhibited decreased ROS signal, while *CAV1*-specific shRNA-transfected (H460/shCav-1) cells showed enhanced ROS signal [52]. Interestingly, Pongjit et al. also established stable Cav-1-overexpressing (H460/Cav-1) cells and investigated the role of Cav-1 in modulating the oxidative stress induced by cisplatin. They found that overexpression of Cav-1 generated significantly higher superoxide anion level and could sensitize cisplatin-induced lung carcinoma cell apoptosis. Meanwhile, lung cancer H460 cells transfected with *CAV1*-specific shRNAs exhibited decreased superoxide generation and decreased cisplatin susceptibility [27]. Although these results revealed a heterogeneous outcome of *CAV1* overexpression/silencing to oxidative stress modulation, they revealed the feedback regulation pattern between Cav-1 and oxidative stress.

### 5.3. Modulation Effects of Antioxidants on Cav-1

**Figure 6:**
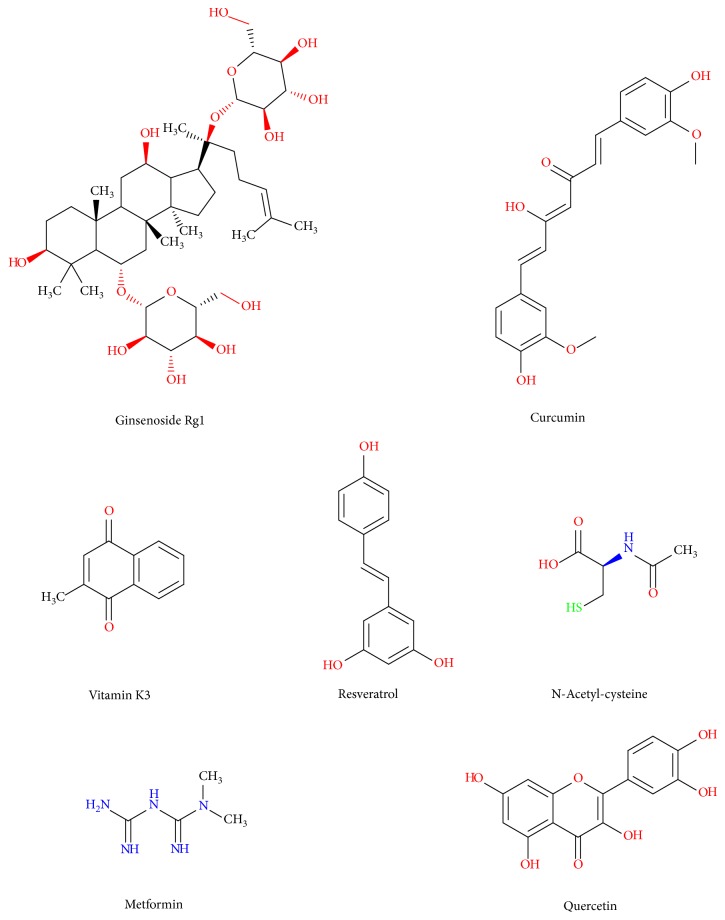
Chemical structures of antioxidants influencing Cav-1 expression.

As described above, reactive species could modulate the expression, degradation, posttranslational modifications, and membrane trafficking of Cav-1, while Cav-1-targeted treatments could scavenge the reactive species. More importantly, emerging evidence has indicated that multiple antioxidants could exert antitumor activities in cancer cells as well as protective activities in normal cells by modulating the Cav-1 pathway (Figure 6).

In terms of cancer cells, Kitano et al. reported that vitamin K3 analogs could induce selective tumor cytotoxicity in neuroblastoma by inducing Cav-1 expression [59]. Yang et al. proved that resveratrol could induce cytotoxicity in a hepatocellular carcinoma model by increasing the expression of endogenous Cav-1 [167]. Salani et al. found that metformin exerted antiproliferative effect in NSCLC cells by inducing the expression of Cav-1 [61]. Martinez-Outschoorn and colleagues reported that treatment with antioxidants (such as N-acetyl-cysteine, metformin, and quercetin) or NO inhibitors was sufficient to reverse many of the Cav-1 loss-induced phenotypes, such as mitochondrial dysfunction, oxidative stress, and aerobic glycolysis in cancer-associated fibroblasts [60].

In terms of normal cells, Zhan et al. reported that ginsenoside Rg1 exerted protective effect on bleomycin-induced pulmonary fibrosis in rats by increasing the expression of *CAV1* mRNA and protein [168]. Nakaso et al. reported that knockdown of Cav-1 and/or Cav-2 prevented the protective effects of tocotrienol in a cellular Parkinson's disease model [169]. In addition, antioxidants such as lycopene [170] and quercetin [171] were also able to induce the expression of Cav-1. In particular, the effects of curcumin on Cav-1 pathway have been deeply reported. For example, Zeng et al. reported that curcumin exerted the antiproliferative effect in airway smooth muscle cells by upregulating the expression of Cav-1, while the disruption of caveolae using methyl-*β*-cyclodextrin attenuated the antiproliferative effects of curcumin [172]. Sun et al. reported that curcumin could prevent EMT of podocytes by suppressing the phosphorylation of Cav-1 and increasing stabilization of Cav-1 and *β*-catenin [173]. In addition, curcumin could inhibit renal inflammatory gene expression in vivo by reducing Cav-1 phosphorylation at Tyr (14) [174]. Yuan et al. investigated the mechanism of curcumin in increasing Cav-1 expression and found that curcumin increased the expression of Cav-1 by inhibiting nuclear translocation of SREBP-1 [175]. In addition, curcumin could suppress ROS overproduction, which was believed to participate in blocking high-glucose-induced apoptosis of podocyte through regulating Cav-1 phosphorylation in both in vitro and in vivo experiments [153]. Altogether, the above evidences suggest that Cav-1 is a target of antioxidants in oxidative stress modulation, further proving that Cav-1 is an oxidative stress-related target for cancer antioxidant prevention.

Although antioxidant treatments have been explored for decades as attractive cancer prevention strategies, no antioxidant treatment regimen has reached the clinic at present. Notably, some preclinical and clinical trials even suggested that the cancer prevention potential of antioxidants was contrary to the expected outcome [176]. For example, Sayin and colleagues recently reported that supplementing the diet with antioxidants N-acetylcysteine and vitamin E markedly increased tumor progression and reduced survival in mouse models of lung cancer [177]. The very large “Selenium and Vitamin E Cancer Prevention Trial” found no initial reduction in the risk of prostate cancer in healthy individuals taking either selenium or vitamin E supplements [178]. Indeed, long-term follow-up studies of these individuals suggested that vitamin E supplementation significantly increased the risk of prostate cancer among healthy men [179]. The disappointing results might be attributed to the multiple targets of antioxidants. Meanwhile, the heterogeneous in the dose, type, schedule, and action mechanisms of different antioxidant drugs might also have great influence on the endpoint. What is more, the poor solubility, stability, and limited bioavailability of some antioxidants also significantly impact on their therapeutic efficacy. Therefore, development of Cav-1-specific targeting antioxidant might be a novel strategy to improve the antioxidant prevention effects.

## 6. Therapeutic Implication of Cav-1-Targeted Treatment for Cancer Antioxidant Prevention

For many years, researchers have theorized that cancer cells depend on the activation of oncogenes or the inactivation of tumor suppressor genes for their survival. This theory is known as “oncogene addiction” [180]. However, recent studies have shed light on the vital mechanisms that ensure the survival of cancer cells, including the ability to escape from immune surveillance as well as the ability to undergo metabolic adaptations that provide cancer cells with a secure energy supply for their uncontrolled proliferation. Thus, targeting the “cart” (e.g., metabolism) rather than the “horse” (oncogenes and tumor suppressor genes) may be a more promising strategy for eliminating cancer cells while sparing normal cells [181]. Cancer cells undergo a metabolism switch, characterized by impaired mitochondrial oxidative phosphorylation [6] and increased aerobic glycolysis [7]. The metabolic reprogramming of malignant cells may be a “cart”, and targeting the aberrant metabolism has been perceived as a more promising strategy for cancer prevention and treatment. As previously stated, aerobic glycolysis could lead to the overproduction of carcinogenic reactive species [7, 182], while Cav-1 could regulate cancer cell metabolism via suppressing MnSOD-driven glycolysis [183]. Meanwhile, Gene microarray analysis revealed that *CAV1*^−/−^ stromal cells showed the upregulation of 48 genes associated with ROS production, 45 genes regulated by HIF-1, and 21 genes involved in glycolysis pathway [184]. In addition, *CAV1*^−/−^ stromal cells exhibited defective mitochondrial functions due to the overexpression of NO [185]. Coculture of *CAV1*^−/−^ stromal cells with cancer cells significantly promoted tumor growth and angiogenesis, while glycolysis inhibitor treatment greatly blocked the positive effects of *CAV1*^−/−^ stromal cells [50, 185]. All these results indicated that Cav-1 may be a critical molecule in linking mitochondrial dysfunction, reactive species elevation, glycolysis enhancement, and finally carcinogenesis promotion. Therefore, therapeutic strategies restoring Cav-1 expression in both cancer cells and adjacent stromal cells should block cancer development via inhibiting glycolytic activity and reactive species overproduction.

On the other hand, compared with normal cells, cancer cells exhibit elevated levels of reactive species. Excessive reactive species not only induce oxidative damages and inactivation of tumor suppressor genes such as *p53* and *PTEN* but also activate the phosphorylation of Cav-1, which is reported to promote cancer cell growth and survival [186]. Tyrosine-14 was documented to be the main phosphorylation site responsible for Cav-1 downstream signaling transduction. Therefore, it is feasible to apply tyrosine kinase inhibitors (TKIs) to interrupt Cav-1 phosphorylation and finally inhibit ROS-induced carcinogenesis. Gefitinib, one kind of TKIs for advanced non-small-cell lung cancer therapy, was reported to inhibit EGF-induced stimulation of both EGFR and downstream Akt and MAPK more efficiently in MCF-7 cells overexpressing Cav-1 than in parental cells [187]. Similarly, the level of phosphorylated Cav-1 was also upregulated in drug-resistant BT474 cells but was blocked by lapatinib [188]. Meanwhile, ovarian cancer cells with high expression of Cav-1 were particularly sensitive to dasatinib [189]. Intriguingly, several TKIs were demonstrated to be effective in preventing carcinogenesis. For example, gefitinib was validated to reduce mammary tumor incidence by 50% [190]. Lapatinib and pazopanib were also demonstrated effective in preventing breast cancer metastasis [191, 192]. What is more is that sorafenib was suggested to be applied for preventing hepatocellular carcinoma recurrence after liver transplantation [193]. All these findings implied that Cav-1 phosphorylation inhibition might be a promising strategy for cancer prevention, and it is of great value to develop Cav-1-targeted TKI for cancer prevention.

The carcinogenic properties of excessive reactive species have prompted the evaluation of dietary antioxidant supplementations as potential cancer preventive agents. However, the preventive efficacies of dietary antioxidant supplementations are disappointing in the current clinical trials. Based on the understanding that mitochondrion is the main source of reactive species in cancer cells, therapies that directly inhibit the production of mitochondrial-derived reactive species, or that scavenge reactive species in mitochondrion, will be more effective than dietary antioxidants, because the latter poorly access the mitochondrial-localized pools of reactive species. Therefore, development of mitochondrion-targeting antioxidants delivery system is a better therapeutic approach for cancer antioxidant prevention. To date, a variety of mitochondrion-targeting antioxidants are being developed, and the best characterized one is 10-(6′-ubiquinonyl) decyltriphenylphosphonium, which could specifically accumulate in the mitochondrial matrix by several hundredfold because of the large mitochondrial transmembrane potential [194]. Caveolae are not the preferred location for Cav-1 in all cell types; instead, Cav-1 can be targeted to a variety of intracellular destinations, particularly in the mitochondrion [21, 22]. *CAV1*-null MEFs (mouse embryonic fibroblasts) had a higher mitochondrial membrane potential and a preference for glycolysis. Meanwhile, the circulating H_2_O_2_ and pyruvate levels in *CAV1*^−/−^ mice were significantly elevated, indicating the altered mitochondrial function and metabolic inflexibility associated with the loss of Cav-1 [195]. Another study also demonstrated that Cav-1 deficiency could increase the mitochondrial membrane condensation, accompanied with dysfunction of respiratory chain efficiency and intrinsic antioxidant defense [196]. Mechanism study further validated that Cav-1 loss resulted in the cytoplasmic and proteasome-dependent degradation of complexes I, III, IV, and V but had no effects on either mitochondrial number or morphology [197]. All these findings suggested that strategies targeting Cav-1 translocation to mitochondrion might be a novel approach for cancer prevention. It is worth to study the detailed molecular mechanisms underlying the targeted delivery of Cav-1 to mitochondrion, and it is anticipated to see enriched mitochondrial Cav-1 expression would decrease the incidence of malignant transformation via inhibiting reactive species overproduction.

## 7. Conclusions and Perspectives

As stated above, both oxidative stress and Cav-1 are closely connected with the tumorigenesis and progression of cancer. Compared with normal cells, cancer cells usually demonstrate aberrations in oxidative metabolism and signaling pathways, characterized by increased levels of reactive species. Reactive species overproduction could induce tumorigenesis and progression possibly by modulating the expression, degradation, posttranslational modifications (including tyrosine phosphorylation and palmitoylation), and subcellular localization of Cav-1. Meanwhile, Cav-1 mainly acts as a tumor suppressor during cancer initiation and development and also has a feedback regulation effect on oxidative stress. More importantly, emerging evidences have indicated that multiple antioxidants could exert antitumor activities in cancer cells as well as protective activities in normal cells by modulating the Cav-1 pathway. Altogether, these observations suggest that Cav-1 may be a potential oxidative stress-related protein for cancer antioxidant prevention. However, it should be noted that existing studies concerning the interaction between Cav-1 and reactive species were mainly applied in normal endothelial or epithelial cells, but less in cancer cells. Therefore, more research focusing on cancer samples are urgent for a better understanding the role of Cav-1 in oxidative stress-induced cancer initiation and progression, as well as its potential as an oxidative stress-related protein for cancer antioxidant prevention. What is more, development of Cav-1-targeting strategies and antioxidant drugs might shed novel light on cancer prevention and antioxidant research.

In summary, this review reveals the roles of Cav-1 and oxidative stress in tumorigenesis and progression as well as their interaction, proposing Cav-1 as a promising candidate target for cancer antioxidant prevention, possibly providing novel insights for Cav-1-targeted strategies for cancer prevention and treatment.

## Figures and Tables

**Table 1:** Caveolin-1 acts as a cancer suppressor in multiple malignancies.

Cancer type	Biofunction	Mechanisms	Reference number
Pancreatic cancer	Cancer suppressor	Inhibiting the EGFR, c-Raf, Mek, and Erk pathways; attenuating the expression of MMPs; inducing apoptosis; and inducing cell cycle arrest in the G0/G1 phase	[30, 90, 91]
Breast cancer	Cancer suppressor	Inducing apoptosis, inducing cell cycle arrest in the G2/M phase, suppressing glycolysis, downregulating the expression of growth factors, inhibiting lysosomal function, inhibiting autophagy, activating the *p53* pathway, inhibiting the AMPK pathway, and inhibiting the Ca^2+^-activated potassium channels	[32, 34–36, 40, 42, 43, 95, 96, 98–101]
Colon cancer	Cancer suppressor	Inhibiting MMP-4 expression, facilitating cyclooxygenase-2, and EGFR degradation	[33, 44, 92, 93]
Melanoma	Cancer suppressor	Suppressing the integrin/Src/FAK pathway	[41, 94]
Leukemia cancer	Cancer suppressor	Inducing apoptosis, inducing the cell cycle arrest in the G1 phase, suppressing the PI3K/AKT/mTOR pathway, suppressing the VEGF redox signal transduction cascades	[82, 84]
Gastric Cancer	Cancer suppressor	Inducing the expression of E-cadherin, inhibiting fibroblast activation, suppressing the Ras/ MAPK pathway	[85–87, 103]
Rhabdomyosarcoma	Cancer suppressor	Suppressing the MAPK pathway, promoting muscular differentiation	[89]
Advanced prostate cancer	Cancer suppressor	Suppressing epithelial Akt activation	[102]
Osteosarcoma	Cancer suppressor	Inhibiting c-Src activity and the Met pathway	[104]
